# Brain gene expression differences related to ethanol preference in the collaborative cross founder strains

**DOI:** 10.3389/fnbeh.2022.992727

**Published:** 2022-09-23

**Authors:** Justin Q. Anderson, Priscila Darakjian, Robert Hitzemann, Denesa R. Lockwood, Tamara J. Phillips, Angela R. Ozburn

**Affiliations:** ^1^Department of Behavioral Neuroscience, Portland Alcohol Research Center, Oregon Health and Science University, Portland, OR, United States; ^2^VA Portland Health Care System, Portland, OR, United States

**Keywords:** central amygdala (CeA), prelimbic cortex (PrL), nucleus accumbens (NAc), genetically unique mouse strains, transcriptomics, ethanol, collaborative cross (CC)

## Abstract

The collaborative cross (CC) founder strains include five classical inbred laboratory strains [129S1/SvlmJ (S129), A/J (AJ), C57BL/6J (B6), NOD/ShiLtJ (NOD), and NZO/HILtJ (NZO)] and three wild-derived strains [CAST/EiJ (CAST), PWK/PhJ (PWK), and WSB/EiJ (WSB)]. These strains encompass 89% of the genetic diversity available in *Mus musculus* and ∼10–20 times more genetic diversity than found in *Homo sapiens*. For more than 60 years the B6 strain has been widely used as a genetic model for high ethanol preference and consumption. However, another of the CC founder strains, PWK, has been identified as a high ethanol preference/high consumption strain. The current study determined how the transcriptomes of the B6 and PWK strains differed from the 6 low preference CC strains across 3 nodes of the brain addiction circuit. RNA-Seq data were collected from the central nucleus of the amygdala (CeA), the nucleus accumbens core (NAcc) and the prelimbic cortex (PrL). Differential expression (DE) analysis was performed in each of these brain regions for all 28 possible pairwise comparisons of the CC founder strains. Unique genes for each strain were identified by selecting for genes that differed significantly [false discovery rate (FDR) < 0.05] from all other strains in the same direction. B6 was identified as the most distinct classical inbred laboratory strain, having the highest number of total differently expressed genes (DEGs) and DEGs with high log fold change, and unique genes compared to other CC strains. Less than 50 unique DEGs were identified in common between B6 and PWK within all three brain regions, indicating the strains potentially represent two distinct genetic signatures for risk for high ethanol-preference. 338 DEGs were found to be *commonly different* between B6, PWK and the average expression of the remaining CC strains within all three regions. The *commonly different* up-expressed genes were significantly enriched (FDR < 0.001) among genes associated with neuroimmune function. These data compliment findings showing that neuroimmune signaling is key to understanding alcohol use disorder (AUD) and support use of these 8 strains and the highly heterogeneous mouse populations derived from them to identify alcohol-related brain mechanisms and treatment targets.

## Introduction

The Collaborative Cross (CC; Collaborative Cross Consortium, 2012) was originally envisaged as a very large panel of mouse recombinant inbred (RI) strains (>1,000 strains) that would make possible the fine mapping of genes relevant to human disorders. The eight founder strains were chosen through a community effort (Churchill et al., 2004) and included five classical inbred laboratory strains [129S1/SvlmJ (S129), A/J (AJ), C57BL/6J (B6), NOD/ShiLtJ (NOD), and NZO/HILtJ (NZO)] and three wild-derived strains [CAST/EiJ (CAST), PWK/PhJ (PWK), and WSB/EiJ (WSB)]. The strains were chosen “to minimize unpredictable genomic interactions between strains while optimizing the genomic contributions of all strains” (Zheng et al., 2015). This was an attempt through a novel breeding strategy to deal with the frequent observation that mouse models that depend on the use of a single strain (often the B6 strain) or a frequently used two-strain cross [often B6 × DBA/2J (D2)] lack the amount of genetic diversity characteristic of human populations.

For a complex set of reasons, the very large panel of CC RI strains was not developed, although there are ∼ 100 CC strains that have proven useful in a variety of contexts (Schoenrock et al., 2020; Bagley et al., 2021; Hackett et al., 2022; Scoggin et al., 2022; Tryndyak et al., 2022). However, the CC founder strains were interbred to establish an outbred population: the heterogeneous stock-collaborative cross (HS-CC; Iancu et al., 2010). Subsequently, and using early generation CC-RI strains, the Diversity Outbred (DO) population was created (Churchill et al., 2012). The differences between the HS-CC and DO populations in breeding histories and genetic features, including the distortion of allele frequencies on chromosome 2 caused by a novel meiotic drive locus, are discussed in Chesler et al. (2016). The breeding of the HS-CC began in 2005. At generation 3, the first complete cross generation, it was observed in a two-bottle choice voluntary alcohol (ethanol) vs. water drinking procedure, that 20% of the population had an ethanol: water preference > 0.5 (unpublished observation); this preference was 2–3 times greater than that found in another heterogeneous cross, the HS/NPT (Crabbe et al., 2012) that was derived from 8 classical inbred laboratory strains of mice including the AJ and B6 strains, but no others shared in common with the CC progenitor strains (see Hitzemann et al., 1994 for cross details). The high HS-CC preference was still present in 2012, when a selective breeding study for high and low ethanol preference was initiated (see Figure 1 in Colville et al., 2017). Colville et al. (2017) observed that selection targeted a network co-expression module that was significantly enriched in genes associated with receptor signaling activity and included *Chrna7, Grin2a, Htr2a*, and *Oprd1.* Connectivity in the module measured by changes in the hub nodes was significantly reduced in the low preference line. Each of the receptor genes had a demonstrated association with alcohol use disorder (AUD) (see references in Colville et al., 2017).

A significant step forward in understanding these and related data (Colville et al., 2018) was made by Bagley et al. (2021). These authors detailed ethanol preference and consumption in the CC founder strains and a subset of the CC RI strains. They observed that the highest ethanol preference, as well as consumption, particularly in females, was in the PWK strain. Preference and consumption exceeded those in the B6 strain, commonly studied for its high ethanol intake. Bagley et al. (2021) additionally demonstrated that strain differences emerge very early in drinking access and have a heritability above 0.8. Alcohol-induced transcriptional regulation is therefore not likely the only driver of genetic influence on drinking. Presented here is transcriptional data from alcohol naive mice from all 8 founder strains that will serve as a bridge between these behavioral data and the data for the selected lines described above. These data will also enable the investigation of potentially critical gene expression differences that precede alcohol exposure. Importantly for this type of analysis, the neurocircuitry and indeed the neurobiology of excessive ethanol and other addictive drug use appears to be very similar in animal models and humans (Koob and Volkow, 2016).

Here, we focus on three regions of the brain ethanol and drug abuse-related circuitry: the central nucleus of the amygdala (CeA), the nucleus accumbens core (NAcc), and the prelimbic cortex (PrL). Of the three regions, the CeA is perhaps most closely aligned with the regulation of ethanol preference (Dhaher et al., 2008), and has a key role in the all 3 stages of addiction (Koob and Moal, 1997; Koob and Volkow, 2010, 2016; Borrego et al., 2022a,b).

## Materials and methods

### Animals

Three male and female mice from each of the 8 CC founder strains (AJ, 129, B6, CAST, NOD, NZO, PWK, and WSB) were obtained from The Jackson Laboratory (Sacramento, CA) and housed within the Veterans Affairs Portland Health Care System (VAPORHCS) Veterinary Medical Unit, an AAALAC approved facility. Mice were group-housed by strain in polycarbonate or polysulfone cages with wire cage tops and ecofresh bedding, and fed standard rodent chow (Purina 5001, PMI Nutrition International, Brentwood, MO, USA). Food and water were available *ad libitum*. The rooms were maintained at 22 ± 1^°^C on a 12:12 h light:dark cycle (lights on at 0600). Animal use and care were approved by the Institutional Animal Care and Use Committee at the VAPORHSC and were in compliance with NIH and USDA guidelines. Animals were acclimated to the new environment for 2–3 weeks prior to euthanasia.

### Tissue

Adult mice (10–12 week old) were euthanized between 10 a.m. and 2 p.m.; brains were removed and immediately frozen on dry ice. To obtain tissue punches from regions of interest, frozen brains were slightly thawed and dissected by hand (under RNAse-free conditions) using a mouse brain atlas as a reference (as in Colville et al., 2018 for PrL and CeA, and Colville et al., 2017 for NAcc). From the anterior aspect, a 1-mm coronal slice of brain tissue was isolated and a blunted 18 gauge needle was used to punch along the midline to obtain prelimbic cortical samples. Moving in a caudal direction, another 1 mm coronal section was obtained using a razor blade, and a blunted 25 gauge needle was used to punch through the section around the anterior commissure to obtain the NAcc. Continuing toward the posterior aspect of the brain, a third 1 mm coronal section was obtained. From this, a blunted 27 gauge needle was used to punch out the CeA. Punches were ejected into empty, RNase free microcentrifuge tubes and stored at –80^°^C until transferred to the Oregon Health and Science University (OHSU) Gene Profiling Shared Resource for RNA isolation.

### RNA-seq

Stranded libraries formation (polyA+) and sequencing were all performed according to Illumina’s specifications at the OHSU Massively Parallel Sequencing Shared Resource (*n* = 144; where *n* = 3/sex/strain/region). Briefly, libraries were prepared using the TruSeq Stranded mRNA kit (Illumina, San Diego CA, USA) and sequencing was done on Illumina’s NovaSeq 6000. A total of 144 libraries were multiplexed in three batches of 48, with each batch balanced for sex, strain and brain region, yielding approximately 25 million total paired-end reads per sample. Sequence alignment was based on the mm10 version of the mouse genome (Ensembl_Mouse_GRCm39_GCA_000001635.9). FastQC (Andrew, 2010) was used for quality checks on the raw sequence data and revealed one batch with significantly lower read counts. This batch was re-sequenced for quality control, bringing the mean counts up to the numbers found in the other two batches. Sequenced data were aligned using the STAR aligner (Dobin et al., 2013), allowing for a maximum of three mismatches per 100 bp read. On average, around 80% of the reads were uniquely aligned. Read counts were also obtained through the STAR aligner. Reads were aligned to the Mus_musculus.GRCm39.105 mouse annotation to generate counts at the gene level. Gene expression data were imported into the R application environment (R Core Team, 2021).

### Analysis

#### Data cleaning

One hundred and forty-four samples (3/sex/strain/region), with counts for 55,320 aligned genes and gene-like features, were analyzed in RStudio using R version 4.1.2. The average coefficient of variation across genes using RNA Seq is relatively small, thus, at *p* < 0.05 and 80% power one can easily detect differences of twice the standard deviation with an N of 6 in each group. Prior to analysis, 40,629 genes were excluded (40,478 for having mean counts-per-million below 1 across samples; 57 for having a count above 50,000 in a single sample; and 94 for being non-chromosomal). Additionally, one sample (an NAcc sample from a female B6 mouse) was excluded as an outlier for having an inter-sample-correlation 5.3 standard deviations from the mean. The biotypes of the 14,785 genes considered during the analyses are found in Supplementary Table 1, along with their annotations; 13,505 (91.3%) were protein coding. The genes dropped from analyses are found in Supplementary Table 2, ∼20% of these genes are protein coding.

#### Principal component analysis

Principal component analysis was performed through the *prcomp* function of R’s base stats package. Principal component analysis (PCA) prior to normalization indicated ∼25% of total explained variance was associated with batch, and ∼58% of total explained variance was associated with brain region and ∼6% was associated with strain. No significant differences in count totals, mean or median were detected between sex or strain. Significant differences were observed between batch and brain region, consistent with the PCA (see Supplementary Figure 1). Counts were normalized using the “trimmed mean of M-values” method (TMM) prior to differential expression (DE) analysis (Robinson and Oshlack, 2010). Post normalization, batch was no longer associated with the first 10 principal components (PC) and the only significant differences in normalized count totals, mean or median were between brain regions (see Supplementary Figure 2).

#### Differential expression

Differentially expressed genes (DEGs) were determined using the limma + voom version 3.52.2 pipeline in edgeR version 3.38.1 (Robinson et al., 2010; Law et al., 2014; Ritchie et al., 2015; Phipson et al., 2016). TMM normalized counts were fit separately for each brain region using strain, sex and batch. Contrasts among strains were used to determine DEGs for each of the 28 possible pairwise comparisons, and for comparing the high and low preference strains (PWK and B6, independently, vs. AJ, CAST, NOD, NZO, S129, WSB). A false discovery rate (FDR), determined *via* the Benjamini-Hochberg methodology (Benjamini and Hochberg, 1995), below 0.05 was used to determine whether a gene was DE for a given comparison, and an absolute log fold change of 1 or higher was used to designate a gene as being a highly DEG (hDEG) for visualization. Statistics were calculated using the *eBayes* and *topTable* functions for FDR cutoffs and the *treat* and *topTreat* functions for log fold change cutoffs. Multiple comparisons of the number of DEGs across contrasts were determined using the *decideTests* function with the option method = “global” following best practices of the limma user guide. The method = “global” option provides the lowest statistical power of *decideTest* methods by treating a set of contrasts as a single test.

#### Visualization

All figures were produced using R. The authors were particularly inspired by the standards and best practices established in the *ggpubr* and *ggstatsplot* packages (Kassambara, 2020; Patil, 2021). The pairwise, multifactor visualizations presented in Figures 2–4 were directly inspired by the data visualization work from Micah Blake McCurdy of *http://hockeyviz.com.*

**FIGURE 1:**
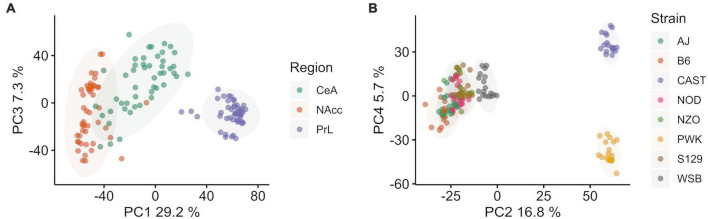
Principal component (PC) analysis of Collaborative Cross founder strains colored by brain region (left, **A**) and strain (right **B**). **(A)** Principal component 1 vs. 3 colored by brain region shows ∼40.1% of total explained variance is associated with brain region. The prelimbic cortex (PrL) is separated from the central nucleus of the amygdala (CeA) and the nucleus accumbens core (NAcc) **(B)** Principal component 2 vs. 4 colored by strain shows ∼37.1% of total explained variance is associated with strain. Wild-derived strains PWK and CAST are isolated in both PC2 and PC4, while WSB and classical inbred laboratory strains are clustered in the middle. Wild-derived strain names are colored purple for clarity. Ellipses represent the 90% confidence interval for each group, assuming a multivariate normal distribution, and are intended as a visual guide.

**FIGURE 2:**
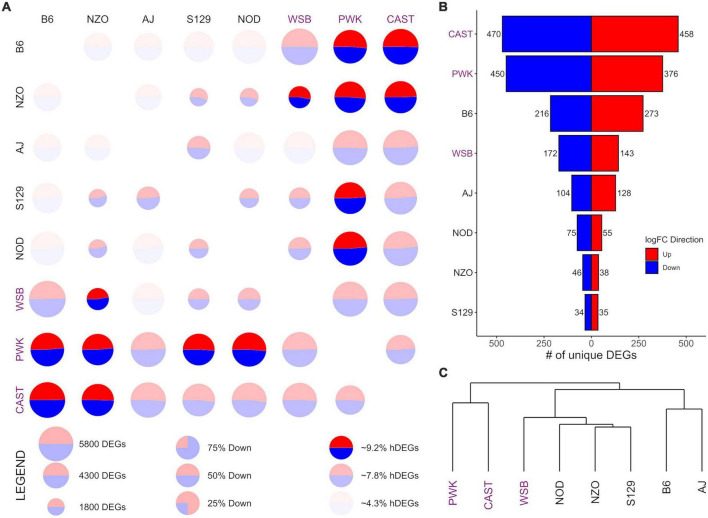
Differential expression (DE) for central nucleus of the amygdala (CeA) samples from the Collaborative Cross founder strains. **(A)** Pairwise DE for founder strains where comparisons are made across a row (e.g., B6 vs. CAST is the upper right circle). For each comparison, the visualization shows: (1) CIRCLE SIZE—corresponding to the total number of differentially expressed genes (DEGs) (FDR < 0.05); (2) CIRCLE COLOR—the ratio of blue (down) to red (up) corresponding to the ratio of down to up DEGs; (3) CIRCLE OPACITY—corresponds to the number of highly DEGs (hDEG; log fold change > 1), where the upper quartile are the brightest, the lower quartile are the dimmest. **(B)** The number of down (blue) and up (red) unique DEGs between a given strain and all others. **(C)** A phylogeny of the founder strains generated *via* hierarchical clustering using Manhattan distance on log fold change. Wild-derived strains PWK, CAST and WSB, denoted by purple text, have the highest number of hDEGS **(A)**, and unique DEGs **(B)**. Among the classical inbred laboratory strains, the B6 demonstrated the highest differentials for hDEGs and unique DEGs. B6 and AJ clustered separately from the remaining classical inbred laboratory strains and WSB, while PWK, and CAST were clustered separately from all other strains **(C)**.

**FIGURE 3:**
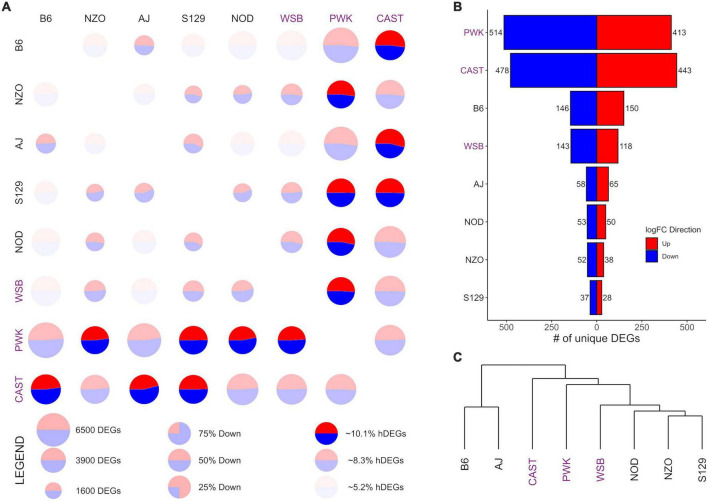
Differential expression (DE) for nucleus accumbens core (NAcc) samples from the Collaborative Cross founder strains. Wild-derived strains PWK, CAST and WSB, denoted by purple text, have the highest number of DEGs, hDEGs **(A)**, and unique DEGs **(B)**. B6 demonstrates the highest differentials for total, hDEGs and unique DEGs among the classical inbred laboratory strains. B6 and AJ cluster separately from all other strains in hierarchical clustering **(C)**.

**FIGURE 4:**
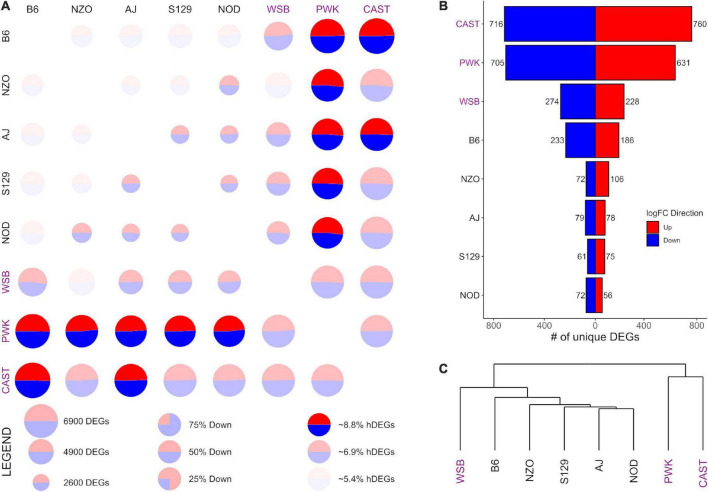
Differential expression (DE) for prelimbic cortex (PrL) samples from the Collaborative Cross founder strains. Wild-derived strains PWK, CAST, and WSB, denoted by purple text, have the highest number of DEGs, hDEGs **(A)**, and unique DEGs **(B)**. B6 demonstrates the highest differentials for total, hDEGs and unique DEGs among the classical inbred laboratory strains. PWK and CAST cluster separately from the other WSB-derived strains in hierarchical clustering **(C)**.

## Results

### Differences among strains are similar to the differences across region

After data cleaning and outlier removal, dimensionality reduction was carried out using PCA to access data quality and the signal to noise ratio for the categorical variables of interest: brain region and mouse strain. PCA was done on the covariance matrix of all 143 samples across 14,785 genes. Brain region was found to be associated with PC 1, 3, and 7, which explained ∼40% of the total variance. Mouse strain was associated with components 2, 4, 5, 6, 8, 9, with a total explained variance of 37.2%. Overall, explained variance in the first 10 components was ∼80%. Sex was not associated with any of the top 10 PC.

PC 1 (29.2% of explained variance) and 3 (7.3% of explained variance) are plotted in Figure 1A, where individual data points are shown and color corresponds with brain region. The PrL appeared to be distinct from the NAcc and CeA, which had some overlap. PC 2 (16.8% explained variance) and 4 (5.7% explained variance) are plotted in Figure 1B, where individual data points are shown and colors now correspond to mouse strain. Wild-derived strains PWK and CAST were separated from WSB and the classical inbred laboratory strains in both components. While the overall variation associated with strains and brain region are similar, we note that a majority of the variance associated with strain separates the wild derived strains PWK and CAST from the classical inbred laboratory strains. The separation of PWK and CAST from one another along PC4 is associated with 3x less explained variance than the separation of PWK and CAST from the other strains along PC4.

### B6 is distinctly different from other classical inbred laboratory strains, while wild-derived CAST and PWK are the most different overall

Given that brain region was associated with a significant share of the total explained variance, DE analysis was conducted for each brain region separately. Comparisons for each of the 28 possible pairwise comparisons between the 8 founder strains within-brain region were carried out, yielding 84 total sets of DEGs. These results are summarized in Figures 2–4 for the CeA, NAcc, and PrL, respectively. For brevity, the visualizations are described in detail only for the case of the CeA, immediately below, and that discussion is not repeated for the results from the NAcc or PrL. For the results presented here, a DEG is defined as having a FDR of less than 0.05, and a *highly differentially expressed gene* (hDEG) has both a FDR rate of less than 0.05 AND an absolute log2 fold change greater or equal to 1, corresponding to either an increase in expression of at least 100% or a decrease in expression of at least 50%. A *unique DEG* is defined as a gene which is differentially expressed between one strain and each of the remaining 7 founder strains in the same direction.

Pairwise CeA DE comparisons are visualized in Figure 2 and summarized in Supplementary Table 3. Figure 2A shows, for each pairwise comparison, the (1) total number of DEGs as the relative size of the circle, (2) the relative number of hDEGs as the color intensity, where the brightest colors correspond to the upper quartile and the dimmest to the bottom quartile, and (3) the ratio of up to down DEGs is shown by the ratio of red to blue. Here comparisons are to be made across a row such that B6 vs. CAST (where “up” expression means higher counts in the B6 strain) is in the upper right corner of Figure 2A, while CAST vs. B6 (where “up” expression means higher counts in the CAST strain) is in the bottom left corner. The wild-derived strains CAST, PWK, and WSB (strain names highlighted with purple color) had the highest numbers of hDEGs. AJ and B6 were the most different of the classical inbred laboratory strains. On average, the inbred lines had 3,965 total DEGs and 262 hDEGs, whereas the wild lines had 4,773 total DEGs and 395 hDEGs.

Figure 2B shows the number of unique DEGs. The lists of these genes are found in Supplementary Table 4. PWK and CAST have over 800 unique DEGs corresponding to approximately 16% of their total DEGs. WSB had 315 unique DEGs (∼7% of their total DEGs). The B6 strain was the most different among the classical inbred laboratory strains, with 489 unique DEGs (∼10% of total). A visual survey of the unique DEG lists for all 8 strains revealed relatively few neurotransmitter genes and transmitter-associated accessory proteins. Notable exceptions included *Oprm1* (up-expressed on average 83% in the PWK strain), *Dlgap4* (down-expressed on average 19% in the B6 strain) and *Gabra2* (down-expressed on average 75% in the B6 strain). The GOrilla algorithm (Eden et al., 2009) was used to detect ontology enrichments of the unique genes seen in the high ethanol preference strains, B6 and PWK, that were not seen in the other strains. Note that up-expressed and down-expressed unique DEGs were analyzed both jointly and then separately. Only one example of such an enrichment was detected. For the B6 strain, and for the up-expressed unique DEGs, there was a significant enrichment in genes associated with immune response. Genes in the category immune system process (*N* = 38; FDR < 1.7 × 10^–3^) are listed in Supplementary Table 5. Included in this list was *B2m* which was on average up-expressed 71%. Supplementary Figure 3 illustrates the interactions of the immune associated ontologies; dark orange shading denotes the most significant enrichments.

Figure 2C shows a phylogeny chart of the HS-CC founder strains, based on hierarchical clustering using the Manhattan distance of a given pair’s absolute log-fold change. This measure provides an equal weight to all log-fold genes in contrast to Euclidean distance, which would be an additional non-linear weighting (on top of the existing log transformation) toward larger changes. PWK and CAST separated from the remainder of the founder lines. The B6 and AJ strains clustered separately from the other classical inbred laboratory strains, which were grouped near WSB. Overall, the wild-derived strains PWK, CAST, and WSB had the highest number of total hDEGs and unique DEGs, with PWK, and CAST being easily identifiable as the most different. The classical inbred laboratory strains were found, in general, to cluster with their nearest wild-derived strain, WSB.

Figures 3, 4 follow the same pattern as Figure 2, but data is presented for the NAcc and the PrL, respectively, with summary statistics presented in Supplementary Tables 6, 7. As in the CeA, in both the NAcc and the PrL, the largest number of unique DEGs was associated with the CAST and PWK strains, while B6 remains the most distinct of the classical inbred laboratory strains. The ratio of total DEGs to hDEGs and unique DEGs also remained similar across regions. However, the number of DEGs identified within the PrL in all strains (wild average: 5,837, lab average: 4,319) is significantly higher than in either the CeA (see above) or the NAcc (wild average: 4,587, lab average: 3,413). The number of hDEGs and unique DEGs within the PrL were similarly high indicating that the PrL is the region where the HS-CC founder strains show the most variation.

Surveying all the NAcc unique DEGs, no neurotransmitter genes were present among the gene lists with the exception of the B6 strain, where both *Gabra2 and Gabrg1* were down-expressed (on average 67% and 47%, respectively). Within the PrL neurotransmitter unique DEGs were identified within B6, NZO, PWK, and CAST [B6 (*Gabra2* [-64%], *Gabrb2* [-26%], *Gria1* [+32%], *Grik4* [-28%], and *Grm2* [-37%]); NZO (*Grik1* [+32%]); PWK (*Gabra2* [+77%], *Glra3* [-65%], *Npy1r* [-29%], and *Npy5r* [-38%]); CAST (*Gabra5* [+51%], *Htra2* [+29%], and *Oprm1* [-58%])].

GO enrichment analysis for the NAcc and PrL followed the same pattern as for the CeA. The unique genes in both regions are found in Supplementary Tables 8, 9. For the NAcc, significant ontology enrichments were found for 2 strains. NOD up-expressed unique DEGs were enriched in genes associated with the extracellular matrix (FDR < 5 × 10^–2^). PWK down-expressed unique DEGs were enriched in mitochondrial genes (FDR < 10^–3^). For the PrL, significant ontology enrichments were also found for 2 strains. B6 down-expressed unique DEGs were enriched in genes associated with neuron morphogenesis (FDR < 4 × 10^–2^) while B6 up-expressed unique DEGs were enriched in genes associated with RNA processing (FDR < 5 × 10^–3^) and regulation of RNA splicing (FDR < 10^–2^). CAST up-expressed unique genes were enriched in genes associated with organic acid metabolism (FDR < 9 × 10^–3^). The genes associated with all the enriched ontologies are found in Supplementary Table 5.

The data in Figure 3C illustrate that in the NAcc, like the CeA, the B6 and AJ strains are clustered together. However, this was not the case in the PrL (Figure 4C). Here, the classical inbred laboratory strains and the WSB clustered together as did the PWK and CAST strains.

### Immune function is a significantly enriched *common difference* between the high ethanol preference strains (B6 and PWK) and the remaining low ethanol preference strains

The unique DEG signatures discussed above suggest the high drinking strains (B6 and PWK) differed from the other HS-CC founder strains in distinct ways, potentially representing two unique risk signatures for high ethanol preference. Therefore, it is critical to investigate whether there are any *common differences* between the high preference strains relative to the low preference strains that may contribute to ethanol preference. Unique DEGs, however, cannot be used to study such a signature, as their definition precludes the possibility of a gene being DE in the same direction in both the B6 vs. PWK and the PWK vs. B6 comparisons. Instead, the genes DE between B6 (or PWK) and the remaining low drinking strains (AJ, CAST, NOD, NZO, S129, and WSB) were studied and are referred to as signature genes. Explicitly, the B6 signature genes are DE in the same direction between B6 and every founder strain except PWK, and the PWK signature genes are similarly the set of genes which are DE in the same direction between PWK and every founder strain except B6.

**FIGURE 5:**
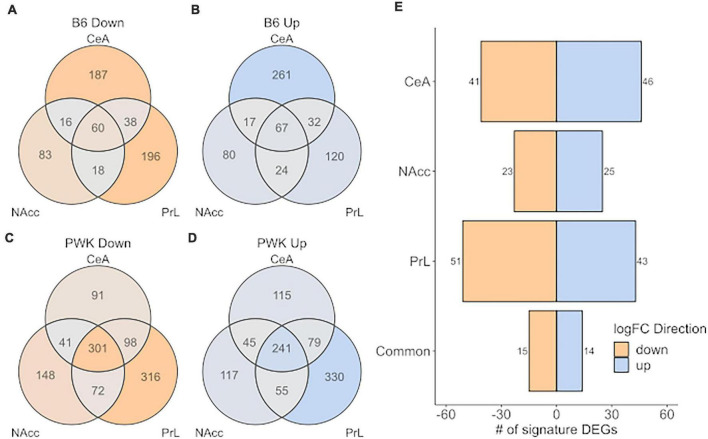
High preference strains vs. low preference strains for unique DEG (strict selection criteria) commonality across strain and brain region. **(A,B)** Identified unique DEGs across individual brain regions for B6. **(C,D)** Identified unique DEGs across brain regions for PWK. **(E)** Unique DEGs *commonly different* between PWK and B6 compared to the low preference strains (AJ, CAST, NOD, NZO, S129, and WSB) for each brain region and in all three brain regions (common). Twenty-nine (15 down- and 14 up-expressed) unique DEGs are found to be *commonly different* between B6 and PWK and the low preference strains across all three brain regions.

Figure 5 shows the number of signature genes for B6 (Figures 5A,B) and PWK (Figures 5C,D) identified for each of the three brain regions and are displayed as a Venn diagram. PWK, the wild-derived strain, had over 3x as many signature genes in common across all three brain regions than did B6 (542 vs. 127). No significant ontology enrichment was identified for either the B6 or PWK strain for these genes common across regions. Figure 5E shows the number of significant genes shared between B6 and PWK in each brain region, as well as in common between all three. Less than 100 genes were identified as common in each individual brain region, and only 29 were shared between all three regions. No significant ontology enrichments were identified using these gene groupings. All the genes identified in common across brain regions in Figure 5 are provided in Supplementary Table 10.

**FIGURE 6:**
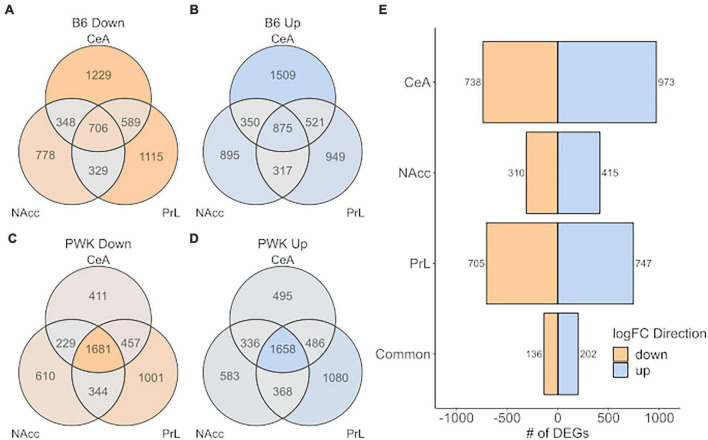
High preference strains vs. low preference strains. DEGs found for B6 and PWK compared the average expression of the low preference strains across strain and brain region (liberal selection criteria; Low_average: (AJ + CAST + NOD + NZO + S129 + WSB)/6). **(A,B)** Identified DEGs across individual brain regions for B6. **(C,D)** Identified DEGs across brain regions for PWK. **(E)** Unique DEGs *commonly different* between PWK and B6 and the low_average for each brain region and in all three brain regions (common). Two hundred and thirty-eight (136 down- and 202 up-expressed) DEGs are found to be *commonly different* between B6 and PWK and the low_average across all three brain regions, more than 10x the number identified using unique DEGs (see Figure 5).

A more relaxed approach for the DE analysis was carried out using the *limma* + *voom* pipeline to compare B6 and PWK, individually, to the *average* expression of the low drinkers [Low_Average: (AJ + CAST + NOD + NZO + S129 + WSB)/6]. As before, genes were considered DE for a given comparison (B6 vs. Low_Average or PWK vs. Low_Average) if the FDR was below 0.05. Note that the signature genes (above) were isolated using pairwise DE results generated by the *limma* + *voom* pipeline, so the two methodologies are completely analogous and differ only in their stringency threshold. This relaxed approach is illustrated in Figure 6 where the DEGs for B6 vs. Low_Average (Figures 6A,B) and PWK vs. Low_Average (Figures 6C,D) identified for each of the three brain regions and are displayed as Venn diagrams. Figure 6E shows the DEGs shared between B6 vs. Low_Average and PWK vs. Low_Average in each brain region. All identified genes in common across brain regions are provided in Supplementary Table 11.

Analysis of the DEGs identified 338 (202 up, 136 down) genes as DE within all three brain regions in both B6 and PWK, over 10x more than identified using the strict signature gene criteria. Note that 100% of the genes identified using the signature genes were also identified by the DE results, confirming the methodologies are consistent and differ only in selection stringency. The 202 up-expressed DEGs in common across all regions (Figure 6E) had significant enrichment in genes associated with immune function (FDR < 10^–3^). An illustration of the immune categories affected is found in Supplementary Figure 4, and the similarity to Supplementary Figure 3 is noted. No significant enrichment was found using the 136 identified down-expressed DEGs. For two reasons we focused on the regionally common DEGs: (1) the B6 and PWK strains are highly distinct from the remaining HS-CC founder strains, and the above requirement limits analysis to genes identified in three independent DE tests, and (2) the genes in common across all three brain regions represent the most likely targets for therapeutic intervention.

## Discussion

The current study is the result of two independent but convergent lines of investigation. The first began with the observation nearly 20 years ago that G3 HS-CC mice had a relatively high ethanol preference (∼20% with a preference for alcohol over water of > 0.5). We note here that in preliminary studies, we have bred mice from 48 pairs of DO parents and obtained essentially the same results (unpublished observation). The HS-CC were allowed to breed for another 20 generations to break up linkages that in turn would facilitate haplotype analyses. The mice were then used to selectively breed for high and low ethanol preference (see Colville et al., 2018, 2017). The goal was to leverage the “high genetic complexity found in HS-CC mice, together with selective breeding, to detect new pathways and mechanisms associated with ethanol preference and excessive ethanol consumption” (Colville et al., 2018). We predicted that molecular signatures of risk for high alcohol intake and preference would provide new targets for therapeutic manipulation (Ferguson et al., 2018; Pozhidayeva et al., 2020). This general argument is described more thoroughly elsewhere in this special issue. The design of Colville et al. (2018) was similar in some respects to the current study in that data were collected across three nodes of the addiction circuit; the CeA, PrL, and NAc shell (rather than the core). The key observation from Colville et al. (2018) was that selection affected across all brain regions a core group of genes that included *Adra1a, Chrna7, Dlg2, Grin2b, Htr2a, Oprd1*, S*str4*, and 17 protocadherins including 14 of the 22 known γ protocadherins. *Dlg2* was considered a key network node in this core grouping (see Figure 1 of Colville et al., 2018). Overall, these data are important because they suggest mechanisms that could distinguish the high and low preference lines, especially mechanisms related to glutamate plasticity. The question that was not answered in Colville et al. (2018) was how the individual CC founder strains contributed to these mechanisms.

The answer to this question was, in part, provided by Bagley et al. (2021), who observed that in addition to the B6 strain, the PWK strain was also a high preference/consumption strain. This observation, while not unexpected, had not been previously explored. Beginning with McClearn and Rodgers (1959), it has been repeatedly found that only the B6 strain and related sub-strains (e.g., Yoneyama et al., 2008) have high ethanol preference and consumption. As noted in Hitzemann et al. (2021) “these data have cast a long shadow on ethanol research resulting in the almost exclusive use of the B6 strain to test for mechanisms of ethanol action and for new therapeutic treatments. This monoculture focus has some obvious advantages including replicability across laboratories and the ability to use genetically modified mice, which are almost exclusively on a B6 or largely B6 background, for hypothesis testing ……the major disadvantage of using the B6 strain ….is that the biology extracted may not be generally applicable, important pathways are missed due to the lack of genetic diversity and individual variation, a key component of some analyses, will be substantially reduced.” Moreover, recent work has shown that B6 mice have global reductions in *Gabra2*, a key subunit of the GABA receptor (an inhibitory ligand gated ion channel important for anxiety-like or alcohol and drug response traits; Mulligan et al., 2019).

The emergence of the PWK strain as a high alcohol preference strain provides new opportunities for hypothesis testing. For example, B6 × D2 genotypes (B6 × D2 crosses, B × D RI strains, B × D congenic strains) there are a core set of studies which have provided consistent genetic information on QTLs and QTGs associated with ethanol preference (e.g., Phillips et al., 1994, 1998; Belknap and Atkins, 2001; Kozell et al., 2020). If the PWK and B6 are contributing the same alleles to high preference, one could predict that a PWK × D2 cross would generate similar QTLs. However, our previous data on multiple cross mapping for other ethanol phenotypes suggests this will be a costly and unsuccessful approach (Malmanger et al., 2006). In the current study, we have taken a different approach—transcriptome profiling the 8 CC founder strains across 3 nodes of the addiction circuit with an emphasis on looking for similarities between the B6 and PWK strains.

The first step in this analysis involved a general survey of the founder strains. The data in Figure 1 confirm an observation first made by Sandberg et al. (2000) that brain regions differ more than strain. The PCA indicated that 40% of the variance was associated with region and only 37% was associated with strain. Sex was not associated with the top 10 Components of the PCA. However, we realize that the analysis was not sufficiently powered to detect sex effects, especially if these were most prominent in only 1 or 2 of the founder strains. In rodents and especially mice (e.g., Bagley et al., 2021), in the choice paradigm, females have a higher ethanol preference. Further, as noted in Hitzemann et al. (2021), sex differences may be associated with differences in gene expression within neuroimmune networks.

The data presented in Figures 2–4 and Supplementary Tables 3–9 indicate the majority of the DEGs involve contrasts between the PWK/CAST and the other 6 founder strains. In terms of generating unique DEGs, the B6 and WSB were relatively equal, less than the unique DEGs generated by the CAST and PWK strains, but significantly more than the unique DEGs associated with the AJ, NOD, NZO, and S129 strains. The unique gene lists were surveyed for neurotransmitter receptors and especially the ligand-gated receptors that are known to be affected by ethanol. In the CeA and NAcc, there was a paucity of transmitter receptors in the unique gene lists. The most notable was the observation that the *Gabra2* subunit is down-expressed in B6 in comparison to the other 7 strains. The role of *Gabra2* in ethanol consumption is discussed in Mulligan et al. (2019). The alignment of transmitter receptors with the unique gene lists was more robust in the PrL and here we remind the reader that from the PCA perspective, the PrL is distinct from the CeA and NAcc. This distinction is not surprising given that the primary neuronal cell types are inhibitory (GABAergic) for both the NAcc and CeA, whereas PrL contains both excitatory and inhibitory neuron cell types. For the B6 strain in the PrL, two GABA (*Gabra2 and Gabrb2)* and three glutamate (*Gria1, Grik4, and Grm2*) receptor genes were detected as unique. For the PWK strain, up-regulation of the *Gabra2* was unique as was the down-regulation of *Npy1r and Npy5r.* The involvement of the neuropeptide Y (NPY) receptor genes in ethanol responses is reasonably well established (e.g., Wetherill et al., 2008; Robinson et al., 2019). Although hardly definitive, these data suggest that from the receptor perspective, the B6 and PWK strains may have a different solution for the mechanisms associated with high preference. We also note that none of these receptor genes associated with the B6 and PWK strains are ones that were detected as aligned with the selection for high and low preference (Colville et al., 2017, 2018), suggesting further that there are many unique molecular signatures of risk for high ethanol preference and consumption.

Ontology enrichment for the unique DEGs (Figures 2–4) clearly suggests that there is no common pattern. For example, one could have predicted that there would be an enrichment in synaptic genes across strains, but the genes associated with the synaptic ontology would differ across strains. This pattern was not observed and in fact, significant ontology enrichments were only rarely observed. For the B6 strain and in the CeA, there was a significant enrichment of genes associated with neuroimmune function. This pattern of enrichment was detected for no other strain. These data align with the now repeated observations that acute and chronic ethanol exposure affects how neuroimmune factors regulate synaptic transmission in B6 mice (Bajo et al., 2014; Patel et al., 2019, 2021; Roberts et al., 2019). Further, the relationship between neuroimmune function and excessive ethanol consumption is well established (Erickson et al., 2019). In the PrL, the B6 unique genes were associated with neuron morphogenesis, cell to cell communication and RNA function, including the regulation of splicing. None of these ontologies were detected for the PWK strain. Rather, there was an enrichment in the NAcc of genes associated with mitochondrial function.

The inclusion of the wild-derived strains as CC founders introduces a statistical problem that can be simply stated as follows: The wild-derived strains have such an excessive amount of DEGs, that mechanisms common to the B6 and PWK, will be simply lost in the PWK signal. To address this issue, we first identified the signature genes (the B6 unique genes w/o the PWK strain and the PWK unique genes w/o the B6 strain) and demonstrated that the two high-preference strains have more signature genes in common than they do signature genes with opposite DE directions, as shown in Figure 5. Next, we performed DE analysis to compare the B6 and PWK, individually, to the remaining 6 low drinking strains and used those results to extract data for the common DEGs across the three brain regions, as shown in Figure 6. Ontology analysis of these data revealed that for the common up-expressed genes, there was an enrichment in genes associated with immune function (see Erickson et al., 2019). These *commonly different* genes were identified as DE, in the same direction, across three brain regions for two potentially distinct genetic signatures for risk for high ethanol-preference (B6 and PWK) and represent a potential target for therapeutic intervention. Pharmacologically targeting pro- and anti-inflammatory immune signaling has indeed been a successful approach for reducing harmful drinking in rodents and in humans (Ferguson et al., 2018; Grigsby et al., 2021). Future work will focus on better understanding how rodent molecular signatures of risk and risk + consequences can result in better harm reduction outcomes for individuals with an AUD diagnosis.

## Data availability statement

The datasets presented in this study can be found in online repositories. Sequencing data as well as raw and normalized counts are available at the Gene Expression Omnibus with accession number GSE212000 (https://www.ncbi.nlm.nih.gov/geo/query/acc.cgi?acc=GSE212000). The analysis script used to produce this manuscript, including figures, is available at the Portland Alcohol Research Center github along with the raw counts and sample metadata needed (https://github.com/parcbioinfo/HSCC_Founders_DE). Curated DE results are available at the github above, and at GeneWeaver. A list of GeneWeaver GeneSets is available at the github.

## Ethics statement

The animal study was reviewed and approved by the Institutional Animal Care and Use Committee at the Veterans Affairs Portland Health Care System Veterinary Medical Unit.

## Author contributions

RH: study design. DL: data collection. JA, PD, and RH: data analysis. JA, RH, DL, PD, TP, and AO: writing and editing the manuscript. All authors contributed to the article and approved the submitted version.

## Supplementary material

Supplementary Figure 1 Distributions (violin plots) of unnormalized total counts per sample, colored and grouped for different factors: **(A)** Sequencing batch, **(B)** Sex, **(C)** Strain, and **(D)** Brain Region. Significant (false discovery rate < 0.05, pairwise *t*-tests) differences in group means were detected between sequencing batches, and between the central nucleus of the amygdala (CeA) and the prelimbic cortex (PrL). Data for all 143 samples are shown.

Supplementary Figure 2 Distributions (violin plots) of trimmed mean of M-values normalized for total counts per sample, colored and grouped for different factors: **(A)** Sequencing batch, **(B)** Sex, **(C)** Strain, and **(D)** Brain Region. Significant (false discovery rate < 0.05, pairwise *t*-tests) differences in group means were detected only between the three brain regions indicating that the batch effect was successfully corrected via normalization. Data for all 143 samples are shown.

Supplementary Figure 3 Enriched ontologies related to immune system process identified using the GOrilla algorithm for up-expressed unique DEGS in the B6 strain in the central nucleus of the amygdala (CeA). No other significantly enriched ontologies were found in the CeA for other strains. Dark orange shading denotes the most significant enrichment.

Supplementary Figure 4 Enriched immune ontologies identified from the *commonly different* DEGs up-expressed between B6 and PWK relative to the average expression of the low preference strains: (AJ+CAST+NOD+NZO+S129+WSB)/6. Dark orange shading denotes the most significant enrichment.
