# Supplementary figures and images for: Brain gene expression differences related to ethanol preference in the collaborative cross founder strains
Source: Front Behav Neurosci. 2022 Sep 23;16:992727. doi: 10.3389/fnbeh.2022.992727 (PMC9539754; doi:10.3389/fnbeh.2022.992727)

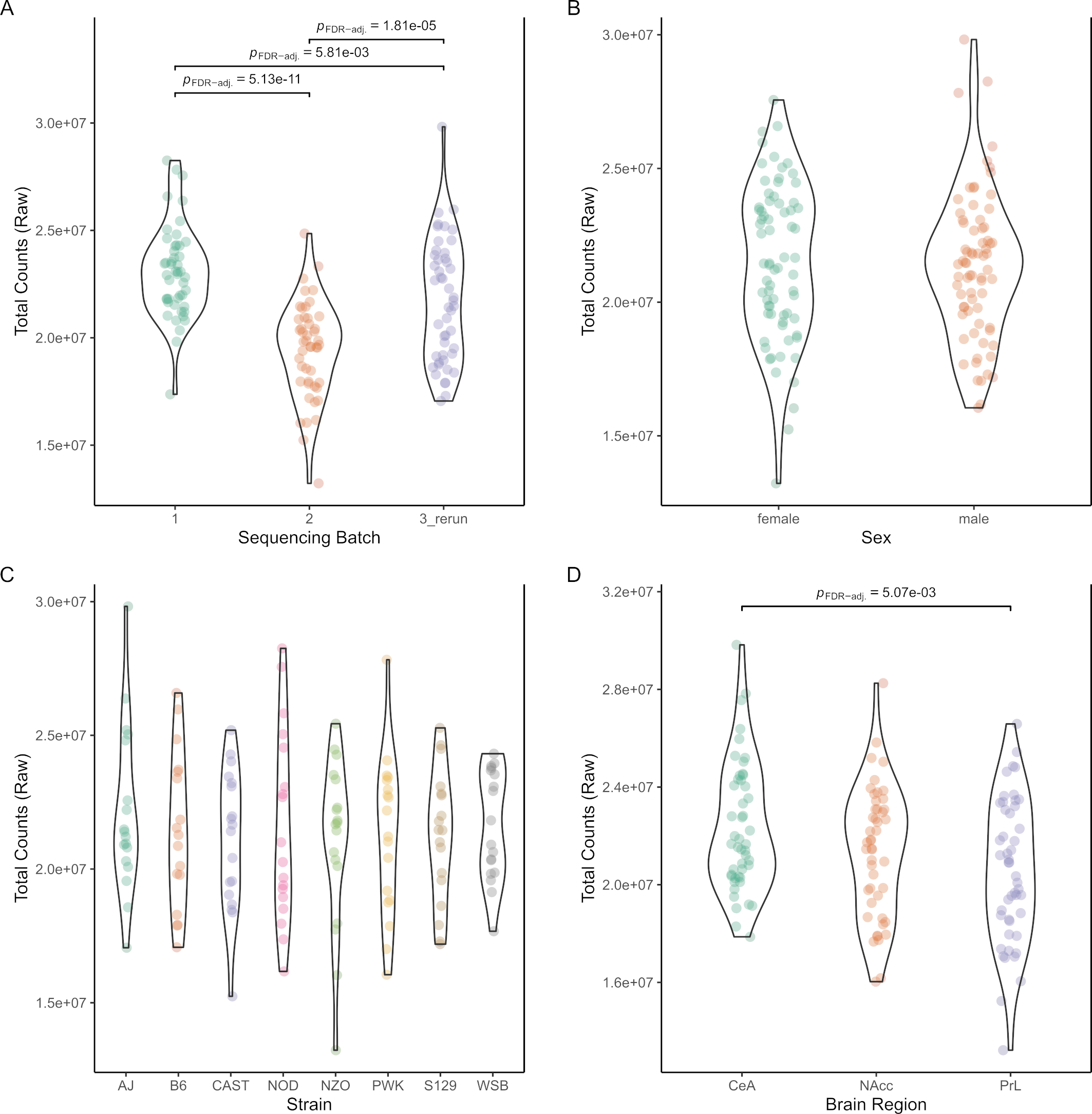

Supplement: Supplementary Figure 1 — Distributions (violin plots) of unnormalized total counts per sample, colored and grouped for different factors: (A) Sequencing batch, (B) Sex, (C) Strain, and (D) Brain Region. Significant (false discovery rate < 0.05, pairwise t-tests) differences in group means were detected between sequencing batches, and between the central nucleus of the amygdala (CeA) and the prelimbic cortex (PrL). Data for all 143 samples are shown. [file Image_1.JPEG]

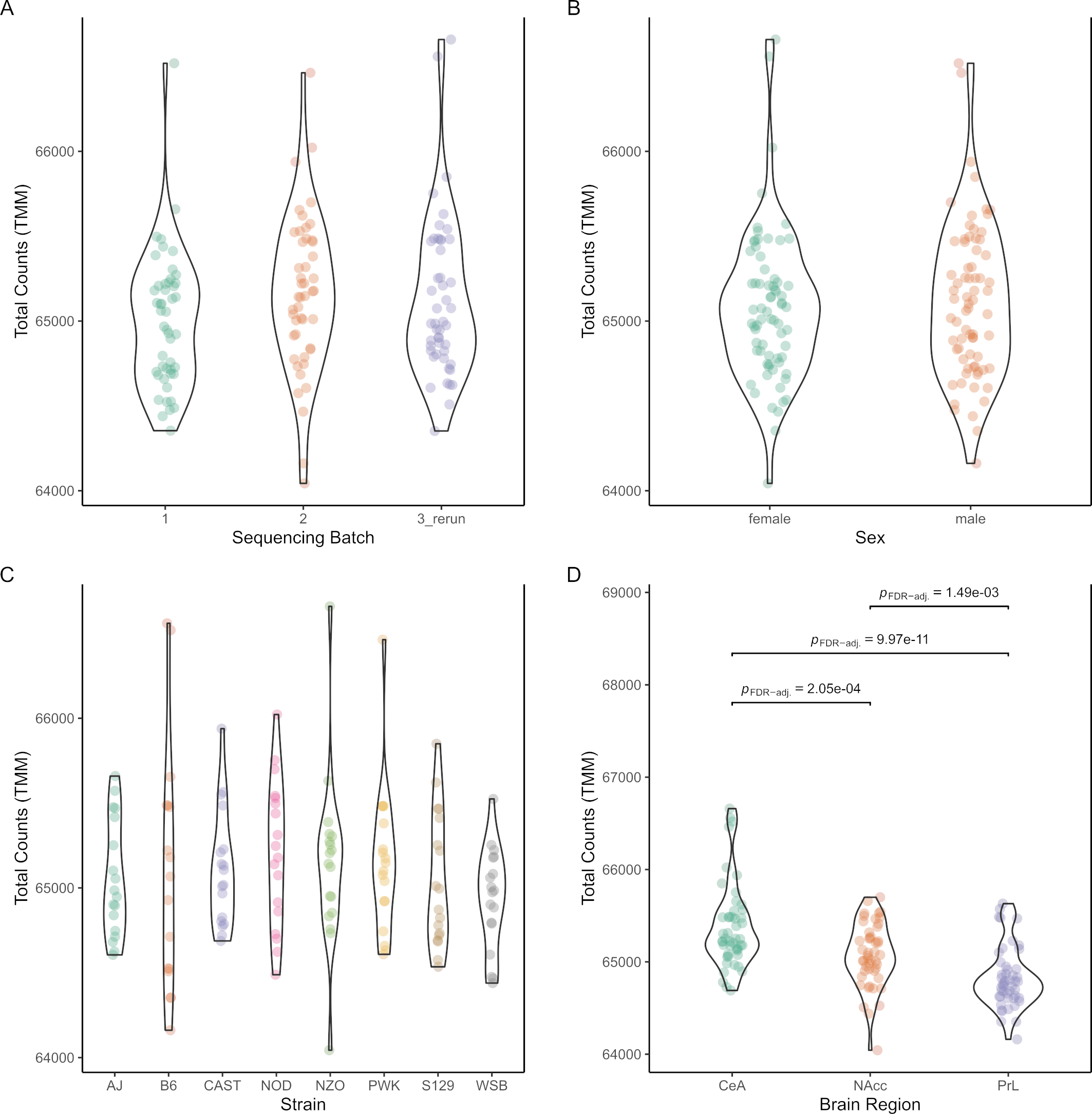

Supplement: Supplementary Figure 2 — Distributions (violin plots) of trimmed mean of M-values normalized for total counts per sample, colored and grouped for different factors: (A) Sequencing batch, (B) Sex, (C) Strain, and (D) Brain Region. Significant (false discovery rate < 0.05, pairwise t-tests) differences in group means were detected only between the three brain regions indicating that the batch effect was successfully corrected via normalization. Data for all 143 samples are shown. [file Image_2.JPEG]

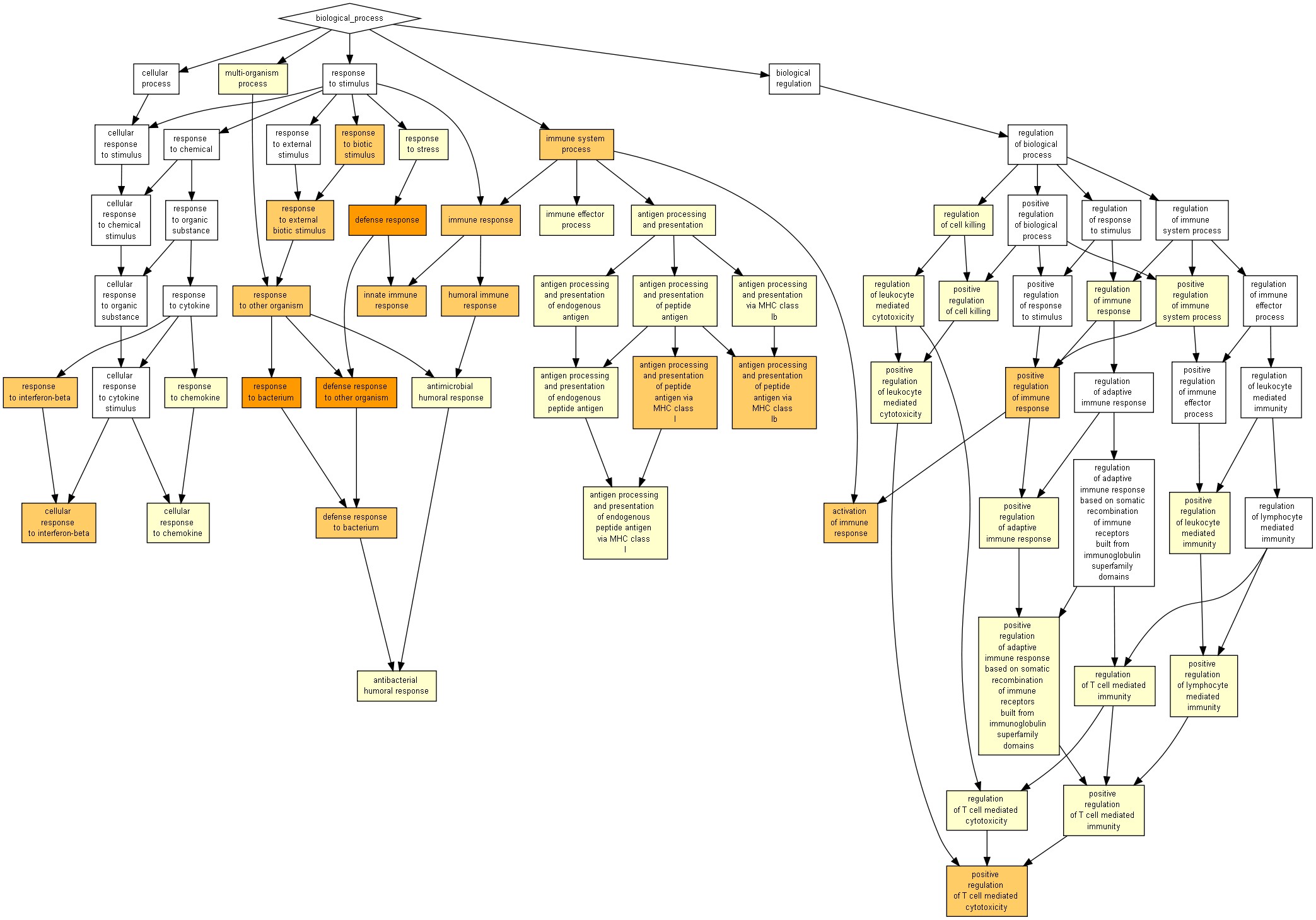

Supplement: Supplementary Figure 3 — Enriched ontologies related to immune system process identified using the GOrilla algorithm for up-expressed unique DEGS in the B6 strain in the central nucleus of the amygdala (CeA). No other significantly enriched ontologies were found in the CeA for other strains. Dark orange shading denotes the most significant enrichment. [file Image_3.JPEG]

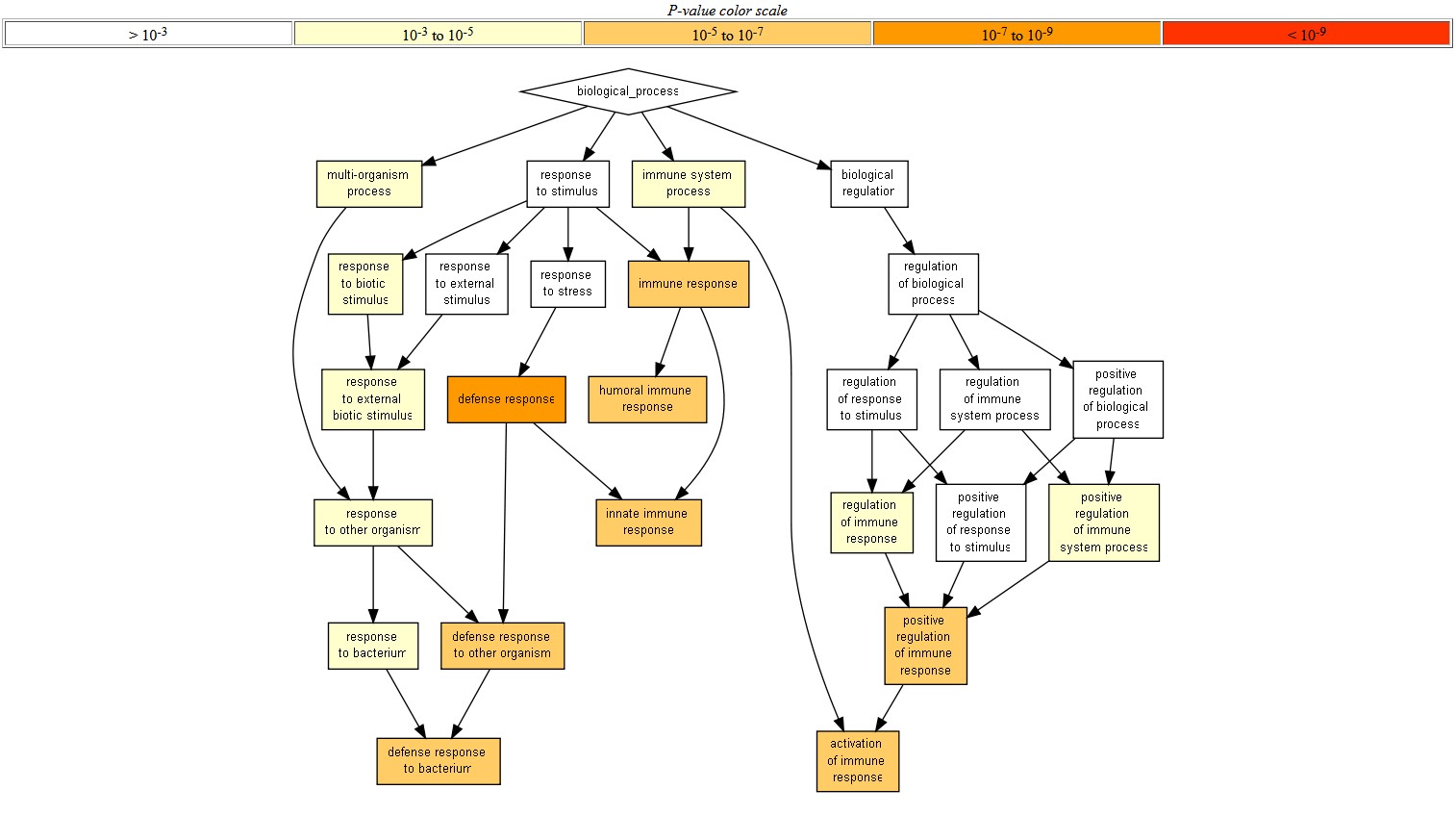

Supplement: Supplementary Figure 4 — Enriched immune ontologies identified from the commonly different DEGs up-expressed between B6 and PWK relative to the average expression of the low preference strains: (AJ+CAST+NOD+NZO+S129+WSB)/6. Dark orange shading denotes the most significant enrichment. [file Image_4.JPEG]
